# Mid‐upper arm circumference is associated with liver steatosis and fibrosis in patients with metabolic‐associated fatty liver disease: A population based observational study

**DOI:** 10.1002/hep4.1990

**Published:** 2022-05-13

**Authors:** Xiaoxiao Wang, Xiaohe Li, Rui Jin, Jia Yang, Rui Huang, Lai Wei, Feng Liu, Huiying Rao

**Affiliations:** ^1^ Peking University Hepatology Institute Beijing Key Laboratory of Hepatitis C and Immunotherapy for Liver Diseases Beijing International Cooperation Base for Science and Technology on NAFLD Diagnosis Peking University People's Hospital Beijing China; ^2^ Beijing Tsinghua Changgung Hospital Tsinghua University Beijing China

## Abstract

Metabolic‐associated fatty liver disease (MAFLD) is a series of liver diseases based on liver steatosis and metabolic disorders. Steatosis, as the core factor in MAFLD diagnosis, and fibrosis, as the major determinant of adverse outcomes of MAFLD, need to be assessed simply and accurately. In this study, we explored the significance of mid‐upper arm circumference (MUAC) in evaluating liver steatosis and fibrosis in patients with MAFLD. We included 2397 cases with MAFLD from the 2017–2018 National Health and Nutrition Examination Surveys (NHANES) database. Liver steatosis and fibrosis were measured by vibration controlled transient elastography. Anthropometric parameters and demographic and serological data were obtained from the NHANES database. The association between MUAC and liver steatosis and fibrosis were evaluated by a multivariable linear regression model, a weighted generalized additive model, and smooth curve fitting using R. MUAC was positively associated with liver steatosis in every multivariate linear regression model (model 1: *β* = 3.3513; 95% confidence interval [CI], 2.7722–3.9304; model 2: *β* = 3.8492; 95% CI, 3.2441–4.4542; model 3: *β* = 2.4987; 95% CI, 1.8371–3.1604), and this positive association was consistent in both men and women and among different race groups (Mexican American, other Hispanic, non‐Hispanic White, Black, Asian, and other race). On the other hand, MUAC was positively associated with liver fibrosis in every multivariate linear regression model, and this positive association also was consistent in both men and women and among non‐Hispanic White and Black populations. Increased MUAC was positively associated with liver steatosis and fibrosis in patients with MAFLD. This was particularly true for MUAC ≥ 42.0 cm. MUAC might be a simple and convenient evaluation tool for MAFLD.

## Graphical abstract

Mid‐upper arm circumference (MUAC) was positively associated with liver steatosis and fibrosis (especially MUAC ≥42cm) in MAFLD patients. As a simply available data during an initial consultation in the outpatient, MUAC might be a simple and convenient tool in MAFLD assessment.

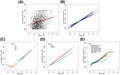

## INTRODUCTION

Nonalcoholic fatty liver disease (NAFLD) is the most common liver disease in the world, accounting for about 25% of the general population.^[^ ^1^, ^2^ ^]^ The epidemiology and pathophysiology of NAFLD are closely connected to a variety of metabolic disorders, including obesity, type 2 diabetes mellitus (T2DM), and cardiovascular disease.^[^ ^3^, ^4^ ^]^ The coexistence of NAFLD and these metabolic factors exacerbates the mortality of NAFLD‐related liver diseases. In 2020, a new concept, metabolic‐associated fatty liver disease (MAFLD), was proposed by international experts and was based on a set of positive diagnostic criteria for fatty liver disease associated with metabolic dysfunction.^[^ ^5^ ^]^ Steatosis, as the core factor in the diagnosis of MAFLD, and fibrosis, as the major determinant of adverse outcomes of MAFLD, need to be accurately assessed by physicians and researchers using concise methods.

In the past, the diagnosis of liver steatosis and fibrosis mainly depended on imaging‐based methods, such as liver vibration‐controlled transient elastography (VCTE) and magnetic resonance elastography. Although these methods are more accurate, specialist doctors are needed for their operation, and patients need to bear the costs for these examinations; these limitations are not helpful for the continuous monitoring and management of patients.^[^ ^6^, ^7^, ^8^ ^]^ This has led researchers to explore more accurate and practical tools for liver assessment. Anthropometric parameters, including the mid‐upper arm circumference (MUAC), can be freely and easily obtained in the outpatient clinic. MUAC is a representative and noninvasive indicator for subcutaneous fat in the upper body, which is often suggested as a novel predictor of nutritional status, central obesity, and insulin resistance (IR).^[^ ^9^, ^10^, ^11^ ^]^ However, there are no published data to evaluate the association between MUAC and liver steatosis and fibrosis. In this study, we included 1640 participants with MAFLD and 757 participants with MAFLD combined with other chronic liver diseases from a national survey database. We explored the association between MUAC and liver steatosis and fibrosis detected by VCTE and evaluated the critical value of MUAC as a screening instrument for MAFLD.

## MATERIALS AND METHODS

### Study population

The 2017–2018 data set was obtained from the National Health and Nutrition Examination Surveys (NHANES) database (https://www.cdc.gov/nchs/nhanes/index.htm) and can be downloaded online for free. NHANES is a cross‐sectional survey conducted by the National Center for Health Statistics (NCHS) of the Centers for Disease Control and Prevention (CDC) in the United States and consists of demographic, dietary, examination, laboratory, and questionnaire data. The NHANES database is widely used in the study of liver diseases. The survey was approved by the CDC ethics review board, and all participants signed informed consent. The study protocol conformed to the ethical guidelines of the 1975 Declaration of Helsinki.

### VCTE

In the NHANES database, only participants in 2017–2018 underwent liver VCTE measurement using the FibroScan 502 V2 Touch (Echosens),^[^ ^12^ ^]^ which is suitable for the study of MAFLD. Controlled attenuation parameter (CAP) and liver stiffness measurements (LSMs) are validated parameters to measure liver steatosis and fibrosis in patients with fatty liver disease.^[^ ^13^, ^14^ ^]^ Liver steatosis was evaluated by the mean of CAP in more than 10 complete operations, and fibrosis was estimated by the mean of LSM in more than 10 complete operations. Individuals without 10 complete FibroScan readings or having an interquartile range (IQR)/median liver stiffness >30% or a fasting time <3 hours were defined as having ineligible FibroScan measurements and were excluded from this study. Significant steatosis was defined as CAP ≥ 248 dB/m, and fibrosis was diagnosed as LSM ≥ 6.3 kPa.^[^ ^14^, ^15^ ^]^.

### Definition of MAFLD

Diagnostic criteria for MAFLD was based on evidence of liver steatosis (data from VCTE measurements in this study) and overweight/obesity (body mass index [BMI] ≥ 25 kg/m^2^) or T2DM (according to international criteria). If patients showed lean or normal weight and did not have T2DM, MAFLD was defined as the presence of liver steatosis and at least two of the following risk factors: (1) waist circumference ≥102 cm in men and 88 cm in women, (2) hypertension (≥130/85 mm Hg or under specific drug therapy), (3) hyperlipidemia (triglyceride [TG] ≥ 1.70 mmol/L or with specific drug treatment), (4) low high‐density lipoprotein cholesterol (HDL‐C) level (<1.0 mmol/L in men and <1.3 mmol/L in women), (5) prediabetes, and (6) hypersensitive C‐reactive protein level >2 mg/L.^[^ ^5^ ^]^ Obese‐MAFLD was defined as patients with MAFLD with BMI ≥ 25 kg/m^2^; nonobese‐MAFLD was defined as patients with MAFLD with BMI < 25 kg/m^2^.^[^ ^5^ ^]^

### Variables

All variables were obtained from the NHANES data set. MUAC, as the independent variable, was measured during the mobile examination center visit, and CAP and LSM, as dependent variables, were determined by VCTE measurement. For covariables, continuous variables included age, anthropometric measures (waist circumference), hip circumference, BMI (waist to hip ratio), fasting plasma glucose (FPG), total cholesterol, triglyceride (TG), high‐density lipoprotein cholesterol (HDL‐C), total bilirubin (TBIL), alanine aminotransferase (ALT), aspartate aminotransferase (AST), γ‐glutamyl transpeptidase, albumin (ALB), alkaline phosphatase, uric acid (UA), hemoglobin A1c (HbA1c), sleep time; categorical variables included sex, race ethnicity (Mexican American, other Hispanic, non‐Hispanic White, non‐Hispanic Black, non‐Hispanic Asian, other race), metabolic diseases (hypertension, diabetes, prediabetes, gout, coronary heart disease [CHD], stroke, thyroid problem), weight loss, medicine (antihypertension medicine, female hormones, and low‐dose aspirin), and ratio of family income to poverty.

### Statistical analysis

All statistical analyses were conducted using R 4.0.2 (http://www.R‐project.org) and EmpowerStats (https://www.empowerstats.net/en/). *p* < 0.05 was considered statistically significant. Sample weights were used to calculate all estimates according to the analytical guideline provided by NCHS. We expressed continuous variables as mean ± SD. Categorical variables were expressed as n (percentages). We constructed three multivariable linear regression models. After stratification by age, sex, and race, analyses based on these three models were further performed in subgroups. To address nonlinearity associations, we also performed a weighted generalized additive model and a smooth curve fitting.

## RESULTS

### Baseline characteristics of participants with MAFLD

**FIGURE 1:**
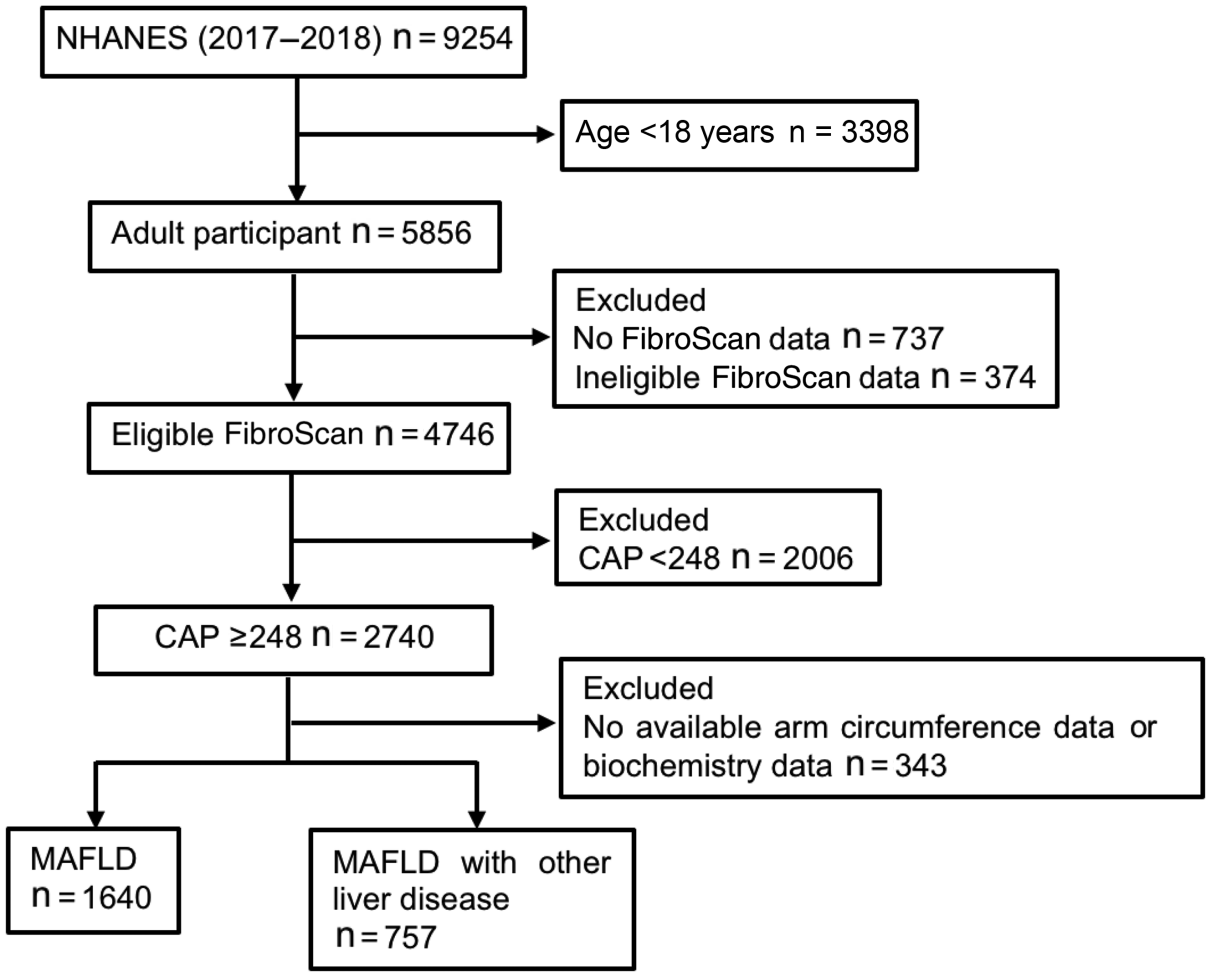
Participant selection flow chart. CAP, controlled attenuation parameter; MAFLD, metabolic‐associated fatty liver disease; NHANES, National Health and Nutrition Examination Survey.

There were 9254 individuals in the 2017–2018 NHANES database. After excluding 3398 cases aged <18 years old, 1111 participants without eligible FibroScan data, 2006 cases with CAP < 248 dB/m, and 343 cases with no available MUAC and biochemistry data, a total of 2397 individuals were applicable for final analysis. All participants were divided into two groups according to whether they had MAFLD (1640 individuals) or MAFLD plus other chronic liver diseases (757 individuals), including heavy alcohol use, chronic hepatitis B, or chronic hepatitis C (Figure 1). The baseline characteristics of participants with MAFLD (n = 1640) are shown in Table 1. Based on a range of BMI values (25–29.9, 30–34.9, 35–39.9, ≥40 kg/m^2^),^[^ ^16^ ^]^ patients with obese‐MAFLD were further divided into four groups. Compared with participants with MAFLD but without obesity, participants with obese‐MAFLD had significantly higher MUAC, waist circumference, hip circumference, waist to hip ratio, liver CAP and stiffness values, UA and HbA1c levels, and percentages of patients with diabetes, prediabetes, CHD, stroke, and trouble sleeping; however, these participants had lower age, HDL‐C, TBIL, ALB, and AST levels. In particular, age, anthropometric parameters, and VCTE parameters increased significantly with the increase in BMI (Table 1). We also analyzed the baseline characteristics of patients with MAFLD with other liver diseases. Except anthropometric and VCTE parameters, individuals with obesity showed higher ALT levels but lower percentages of cases with hypertension, diabetes, gout, and CHD than participants without obesity (Table S1).

**TABLE 1:** Baseline characteristics of patients with MAFLD assessed by VCTE in the NHANES database, 2017–2018

Variables	Nonobese‐MAFLD (n = 125)	Obese‐MAFLD (n = 1515)				*p* value
	BMI (<25 kg/m^2^)	BMI (25–29.9 kg/m^2^) n = 584	BMI (30–34.9 kg/m^2^) n = 471	BMI (35–39.9 kg/m^2^) n = 263	BMI (≥40 kg/m^2^) n = 197	
Age (years)	62.5 ± 12.8	57.1 ± 16.4	55.4 ± 16.8	53.4 ± 16.5	51.4 ± 14.8	<0.001
Sex, n (%)						
Male	72 (57.6)	359 (61.5)	266 (56.5)	122 (46.4)	75 (38.1)	<0.001
Female	53 (42.4)	225 (38.5)	205 (43.5)	141 (53.6)	122 (61.9)	
Race, n (%)						<0.001
Mexican American	7 (5.6)	95 (16.3)	83 (17.6)	37 (14.1)	26 (13.2)	
Other Hispanic	9 (7.2)	73 (12.5)	37 (7.9)	23 (8.7)	12 (6.1)	
Non‐Hispanic White	38 (30.4)	179 (30.7)	175 (37.2)	111 (42.2)	75 (38.1)	
Non‐Hispanic Black	9 (7.2)	81 (13.9)	102 (21.7)	68 (25.9)	58 (29.4)	
Non‐Hispanic Asian	57 (45.6)	131 (22.4)	50 (10.6)	15 (5.7)	8 (4.1)	
Mexican American	5 (4.0)	25 (4.3)	24 (5.1)	9 (3.9)	18 (9.1)	
Anthropometric parameters						
BMI (kg/m^2^)	23.5 ± 1.2	27.6 ± 1.4	32.3 ± 1.4	37.1 ± 1.4	45.1 ± 4.7	<0.001
MUAC (cm)	28.8 ± 1.9	32.1 ± 2.4	35.6 ± 2.6	38.2 ± 3.0	42.5 ± 4.2	<0.001
Waist circumference (cm)	89.5 ± 6.0	98.2 ± 7.1	108.9 ± 7.5	118.5 ± 8.0	132.1 ± 10.2	<0.001
Hip circumference (cm)	94.2 ± 4.2	101.9 ± 5.3	110.7 ± 6.2	121.2 ± 7.3	135.8 ± 10.6	<0.001
Waist to hip ratio	1.0 ± 0.1	1.0 ± 0.1	1.0 ± 0.1	1.0 ± 0.1	1.0 ± 0.1	<0.001
VCTE parameters						
CAP (dB/m)	290.1 ± 33.4	297.1 ± 33.8	308.4 ± 38.8	324.1 ± 42.7	335.6 ± 45.4	<0.001
Stiffness (kPa)	5.3 ± 3.8	5.7 ± 4.7	6.1 ± 4.3	6.7 ± 3.5	9.1 ± 7.5	<0.001
Serum test						
FPG (mmol/L)	6.6 ± 3.0	6.0 ± 2.1	6.1 ± 2.5	6.0 ± 2.1	6.5 ± 2.8	0.037
TC (mmol/L)	5.2 ± 1.2	5.0 ± 1.1	4.9 ± 1.0	4.8 ± 1.1	4.7 ± 1.0	0.001
TG (mmol/L)	2.2 ± 1.3	1.9 ± 1.4	1.9 ± 1.4	1.9 ± 1.1	1.9 ± 1.5	0.233
HDL‐C (mmol/L)	1.3 ± 0.4	1.3 ± 0.3	1.2 ± 0.3	1.2 ± 0.3	1.2 ± 0.3	0.002
TBIL (μmol/L)	8.5 ± 5.6	8.4 ± 4.8	7.9 ± 4.8	7.2 ± 3.8	7.2 ± 3.9	<0.001
ALB (g/L)	41.5 ± 3.2	41.3 ± 3.0	40.3 ± 3.1	39.7 ± 2.9	38.6 ± 2.9	<0.001
ALT (U/L)	22.4 ± 12.9	22.9 ± 13.9	25.3 ± 16.0	26.6 ± 20.8	23.5 ± 13.3	0.010
AST (U/L)	22.2 ± 8.3	21.5 ± 9.9	22.5 ± 11.1	22.7 ± 14.1	20.6 ± 8.5	0.157
GGT (U/L)	36.9 ± 63.4	31.8 ± 30.3	33.7 ± 37.9	32.3 ± 33.5	35.6 ± 32.4	0.525
ALP (U/L)	82.5 ± 29.5	80.5 ± 22.7	81.1 ± 24.5	82.1 ± 23.5	88.0 ± 29.4	0.007
Creatinine (μmol/L)	83.3 ± 56.8	82.0 ± 33.4	85.3 ± 57.9	80.9 ± 61.6	76.6 ± 23.2	0.298
UA (μmol/L)	330.0 ± 84.2	338.7 ± 83.6	341.9 ± 80.3	360.9 ± 95.4	370.7 ± 101.5	<0.001
HbA1c (%)	6.4 ± 1.6	6.1 ± 1.3	6.1 ± 1.2	6.2 ± 1.3	6.4 ± 1.5	0.013
Metabolic diseases						
Hypertension, n (%)	45 (36.0)	147 (25.2)	119 (25.3)	83 (31.6)	62 (31.5)	0.034
Diabetes, n (%)	42 (33.6)	123 (21.1)	113 (24.0)	59 (22.4)	63 (32.0)	0.004
Prediabetes, n (%)	10 (8.0)	65 (11.1)	55 (11.7)	39 (14.8)	30 (15.2)	<0.001
Gout, n (%)	19 (15.2)	41 (7.0)	24 (5.1)	20 (7.6)	18 (9.1)	0.004
CHD, n (%)	13 (10.4)	32 (5.5)	33 (7.0)	14 (5.3)	12 (6.1)	0.170
Stroke, n (%)	6 (4.8)	20 (3.4)	22 (4.7)	20 (7.6)	9 (4.6)	0.076
Thyroid problem, n (%)	13 (10.4)	66 (11.3)	79 (16.8)	39 (14.8)	23 (11.7)	0.047
Sleep time (hours)						
Weekdays	7.8 ± 1.7	7.5 ± 1.6	7.5 ± 1.6	7.5 ± 1.7	7.4 ± 1.8	0.162
Weekends	8.3 ± 1.6	8.2 ± 1.7	8.2 ± 1.7	8.1 ± 1.7	8.1 ± 1.9	0.576
Trouble with sleep, n (%)	28 (22.4)	129 (22.1)	149 (31.6)	98 (37.3)	87 (44.2)	<0.001
Weight loss, n (%)	84 (67.2)	406 (69.5)	326 (69.2)	191 (72.6)	133 (67.5)	0.753
Medicine, n (%)						
Hypertension	45 (36.0)	213 (36.5)	201 (42.7)	118 (44.9)	87 (44.2)	<0.001
Female hormones	14 (11.2)	47 (7.0)	41 (8.7)	27 (10.3)	20 (10.2)	<0.001
Low‐dose aspirin	39 (31.2)	177 (30.3)	158 (33.5)	80 (30.4)	73 (37.1)	0.610
Ratio of family income to poverty						0.005
<1.0	16 (12.8)	69 (11.8)	61 (13.0)	52 (19.8)	40 (10.3)	
1.0 to <2.0	26 (20.8)	148 (25.3)	98 (20.8)	67 (25.5)	55 (27.9)	
2.0 to <3.0	27 (21.6)	82 (14.0)	79 (16.8)	36 (13.7)	36 (18.3)	
3.0 to <5.0	18 (14.4)	111 (19.0)	86 (18.3)	40 (15.2)	25 (12.7)	
≥5.0	19 (15.2)	92 (15.8)	79 (16.8)	36 (13.7)	13 (6.6)	
NA	19 (15.2)	82 (14.0)	68 (14.4)	32 (12.2)	28 (14.2)	

*Note*: Continuous variables are shown as mean ± SD. Categorical values are shown as n (%).

Abbreviations: ALB, albumin; ALP, alkaline phosphatase; ALT, alanine aminotransferase; AST, aspartate aminotransferase; BMI, body mass index; CHD, coronary heart disease; FPG, fasting plasma glucose; GGT, γ‐glutamyl transpeptidase; HbA1c, hemoglobin A1c; HDL‐C, high‐density lipoprotein cholesterol; MAFLD, metabolic‐associated fatty liver disease; MUAC, mid‐upper arm circumference; NA, not available; NHANES, National Health and Nutrition Examination Survey; TBIL, total bilirubin; TC, total cholesterol; TG, triglyceride; UA, uric acid; VCTE, vibration controlled transient elastography.

### Multivariate analysis for MUAC and liver steatosis

Based on the increase of BMI in all our participants, MUAC and liver CAP values showed a similar increasing trend (Figure S1). Multivariate linear regression analysis was further performed to evaluate the association between MUAC and liver steatosis. Three models were used to evaluate *β* (95% CI) of the MUAC. In model 1, no covariates were adjusted. For model 2, age, sex, and race were adjusted. Model 3 was adjusted for additional hypertension; waist to hip ratio; the level of FPG, HDL, ALT, TG, and UA; obesity; and median liver stiffness on the basis of model 2.

**FIGURE 2:**
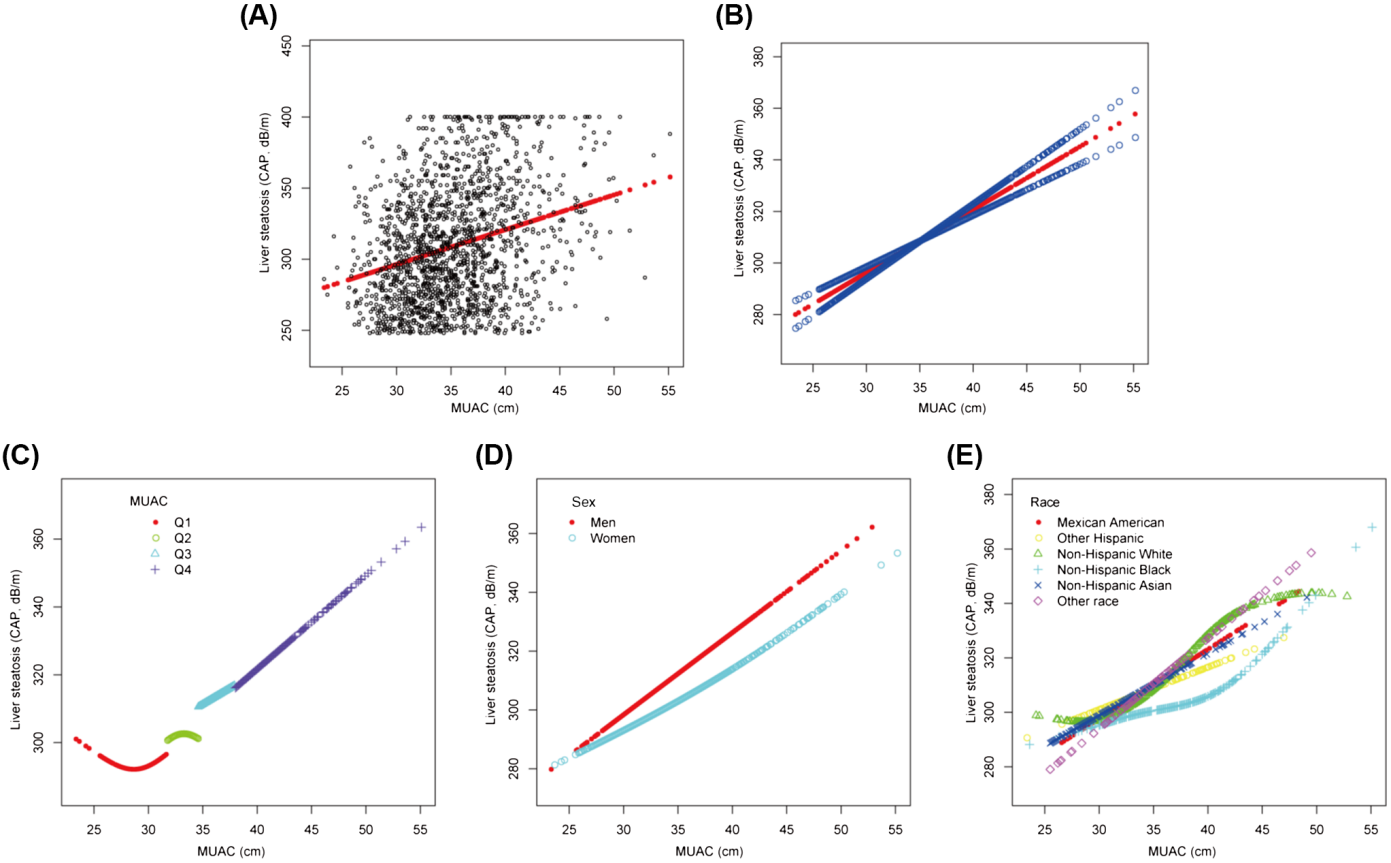
Association between MUAC and liver steatosis (CAP) in patients with MAFLD. (A) Each sample is represented by a black point. (B) The smooth curve fit (red band) and the 95% confidence interval from the fit (blue band) between two variables. Age, sex, race, hypertension, and waist to hip ratio; level of fasting plasma glucose, alanine aminotransferase, high‐density lipoprotein cholesterol, triglyceride, and uric acid; and obesity and median liver stiffness were adjusted. Association between MUAC and liver steatosis in (C) four quartiles of MUAC, (D) men and women, and (E) different racial subgroups. CAP, controlled attenuation parameter; MAFLD, metabolic‐associated fatty liver disease; MUAC, mid‐upper arm circumference; Q, quartile.

The results of the multivariable analysis showed that MUAC was positively associated with the liver CAP value of patients with MAFLD in each multivariable linear regression model (Table 2). This trend remained significant among different MUAC quartile groups (*p* < 0.001). The positive association was also observed in both men (*β* = 3.7379; 95% CI, 2.6379–4.8018; *p* < 0.001) and women (*β* = 1.6706; 95% CI, 0.8381–2.5031; *p* < 0.001) as well as in all racial subgroups (Table 2). We used a weighted generalized additive model and a smooth curve fitting to address the nonlinear relationship and confirmed that liver CAP values increased consistently with the increase of MUAC (Figure 2A–E). Although there were subtle differences, the relationship between MUAC and liver CAP values in patients with MAFLD with other chronic liver diseases was similar to patients with MAFLD (Table S2; Figure S2A–E).

**TABLE 2:** Multivariate analysis for the relationship between MUAC and liver steatosis in patients with MAFLD (CAP)

	Model 1, *β* (95% CI) *p* value (n = 1640)	Model 2, *β* (95% CI) *p* value (n = 1640)	Model 3, *β* (95% CI) *p* value (n = 1640)
Baseline MUAC	2.7620 (2.3661, 3.1580)	3.1050 (2.6884, 3.5216)	2.1389 (1.7021, 2.5757)
	<0.000001	<0.000001	<0.000001
Quartiles of MUAC			
Q1 (22.5–32.0 cm)	Reference	Reference	Reference
Q2 (32.1–34.8 cm)	9.1144 (3.4647, 14.7608)	10.7911 (5.1185, 16.4637)	6.9184 (1.1925, 12.6442)
	0.001586	0.000199	0.017991
Q3 (34.9–38.2 cm)	20.1829 (14.8649, 25.5009)	22.4020 (16.9414, 27.8627)	14.4033 (8.7783, 20.0282)
	<0.000001	<0.000001	<0.000001
Q4 (38.3–56.3 cm)	33.2188 (27.8287, 38.6089)	37.1152 (31.4632, 42.7672)	22.4071 (18.6802, 30.5340)
	<0.000001	<0.000001	<0.000001
*p* for trend	<0.001	<0.001	<0.001
Stratified by sex^a^			
Men	3.3304 (2.7329, 3.9280)	3.8604 (3.2356, 4.4852)	2.3599 (1.6772, 3.0426)
	<0.000001	<0.000001	<0.000001
Women	2.0622 (1.5481, 2.5763)	2.3727 (1.8319, 2.9135)	1.8694 (1.3030, 2.4357)
	<0.000001	<0.000001	<0.000001
Stratified by race^a^			
Mexican American	2.3470 (1.1959, 3.4981)	2.3356 (1.1729, 3.4983)	1.5825 (0.4047, 2.7603)
	<0.000085	<0.000108	0.009015
Other Hispanic	3.3735 (1.7805, 4.9665)	3.8057 (2.1246, 5.4869)	3.2833 (1.4848, 5.0818)
	<0.000055	<0.000018	0.000476
Non‐Hispanic White	2.8995 (2.2309, 3.5681)	3.1294 (2.4313, 3.8214)	1.9706 (1.2625, 2.6788)
	<0.000001	<0.000001	<0.000001
Non‐Hispanic Black	2.3687 (1.4438, 2.2935)	2.7771 (1.8441, 3.7101)	2.5752 (1.5570, 3.5933)
	<0.000001	<0.000001	<0.000001
Non‐Hispanic Asian	2.5474 (1.4685, 3.6263)	3.0230 (1.8277, 4.2184)	1.9308 (0.6167, 3.2448)
	<0.000006	<0.000001	0.004328
Other race	3.4356 (2.0366, 4.8346)	4.2583 (2.6799, 5.8367)	3.1664 (0.8966, 5.4363)
	<0.000007	<0.000001	0.07992

*Note*: Model 1: No covariates were adjusted. Model 2: Sex, age, and race were adjusted. Model 3: Sex, age, race, waist to hip ratio, fasting plasma glucose, high‐density lipoprotein cholesterol, alanine aminotransferase, triglyceride, uric acid, hypertension, obesity, and median liver stiffness were adjusted.

Abbreviations: CAP, controlled attenuation parameter; CI, confidence interval; MAFLD, metabolic‐associated fatty liver disease; MUAC, mid‐upper arm circumference; Q, quartile.

^a^ In the subgroup analysis stratified by sex or race, models 1–3 were not adjusted for their own stratification variable.

### Multivariate analysis for MUAC and liver fibrosis

**FIGURE 3:**
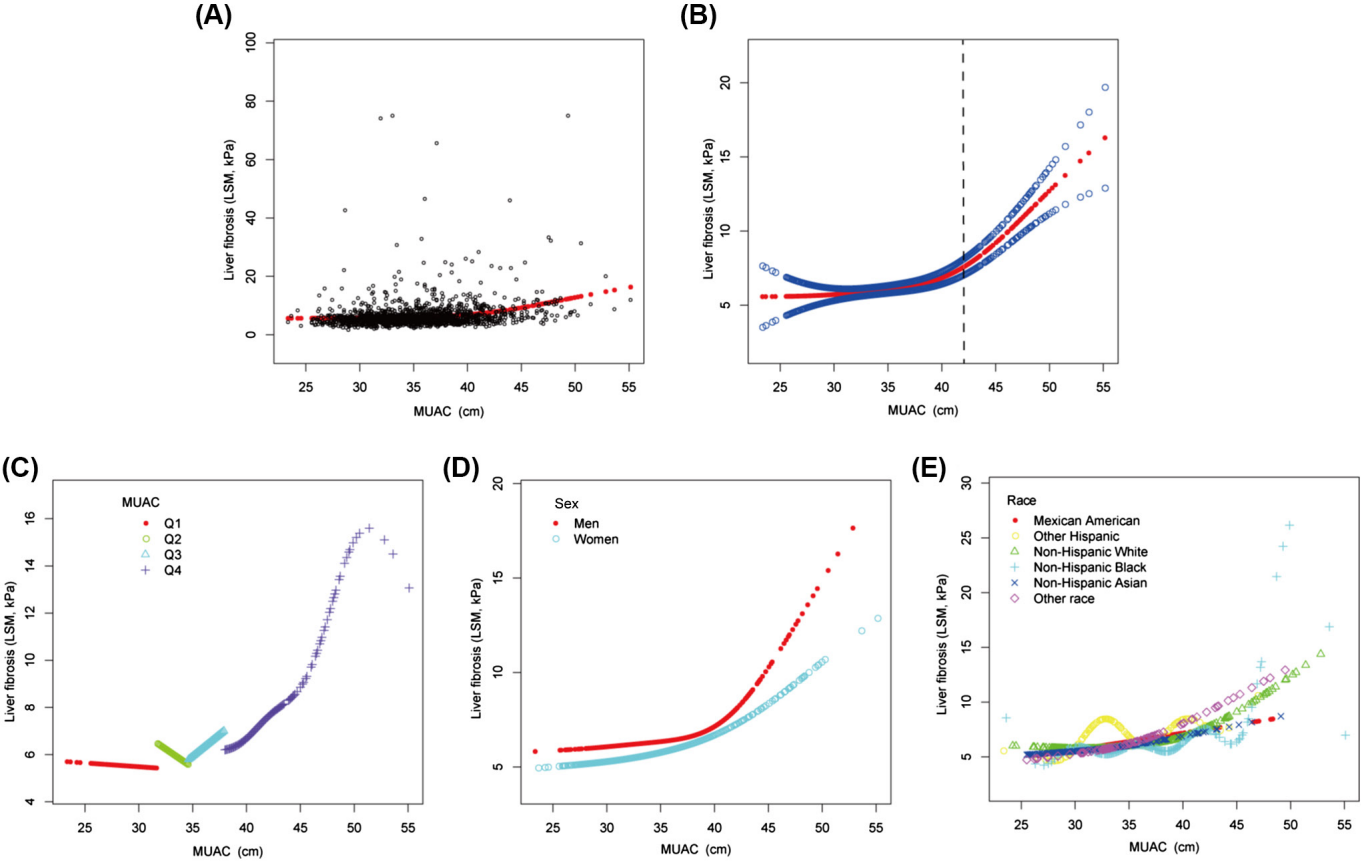
Association between MUAC and liver fibrosis (LSM) in patients with MAFLD. (A) Each sample is represented by a black point. (B) The smooth curve fit (red band) and the 95% confidence interval from the fit (blue band) between two variables. Age, sex, race, hypertension, and waist to hip ratio; level of fasting plasma glucose, alanine aminotransferase, high‐density lipoprotein cholesterol, triglyceride, and uric acid; obesity and median liver CAP were adjusted. Association between MUAC and liver fibrosis in (C) four quartiles of MUAC, (D) men and women, and (E) six racial subgroups. CAP, controlled attenuation parameter; LSM, liver stiffness measurement; MAFLD, metabolic‐associated fatty liver disease; MUAC, mid‐upper arm circumference; Q, quartile.

Similarly, we also observed a simultaneous and increasing trend in MUAC and liver stiffness value with the increase in BMI in all our cases (Figure S1). In this multivariable analysis, model 1 and model 2 were adjusted for the same variables as in liver steatosis. Except for other variables in liver steatosis, model 3 was also adjusted for the median liver CAP value instead of median liver stiffness (Table 3). The positive relationship between MUAC and liver stiffness existed in each model (*p* < 0.001). The positive association was also observed in both men (*β* = 0.3347; 95% CI, 0.1626–0.5068; *p* < 0.001) and women (*β* = 0.5007; 95% CI, 0.3171–0.6842; *p* < 0.001). However, after being stratified by quartiles of MUAC, the positive association only persisted in the fourth quartile (38.3–56.3 cm) in models 1–3. With an increase of MUAC, the positive correlation became more obvious. Moreover, in the subgroup analysis stratified by race, the positive relation between MUAC and liver fibrosis was only present in the non‐Hispanic White and Black populations (*p* < 0.01) (Table 3). A weighted generalized additive model and a smooth curve fitting was also used to illustrate the nonlinear relationship (Figure 3A–E). We found that at MUAC ≥ 42.0 cm (95% CI, 39.3–42.5), MUAC provided a better evaluation parameter for liver fibrosis (Figure 3A,B). In addition, MUAC ≥ 42.0 cm (95% CI, 39.6–42.9) provided a better evaluation parameter for liver fibrosis in men (red line), and MUAC ≥ 42.6 cm (95% CI, 38.9–45.3) provided a better evaluation parameter for liver fibrosis in women (blue line) (Figure 3D). However, in patients with MAFLD plus other liver diseases, the positive relationship between MUAC and liver stiffness was not apparent in women or different racial subgroups (Table S3; Figure S3).

**TABLE 3:** Multivariate analysis for the relationship between MUAC and liver fibrosis in patients with MAFLD (LSM)

	Model 1, *β* (95% CI) *p* value (n = 1640)	Model 2, *β* (95% CI) *p* value(n = 1640)	Model 3, *β* (95% CI) *p* value (n = 1640)
Baseline MUAC	0.2355 (0.1899, 0.2810)	0.2703 (0.2223, 0.3183)	0.2088 (0.1554, 0.2622)
	<0.000001	<0.000001	<0.000001
Quartiles of MUAC			
Q1 (22.5–32.0 cm)	Reference	Reference	Reference
Q2 (32.1–34.8 cm)	0.3572 (−0.2988, 1.0133)	0.4105 (−0.2508, 1.0717) 0.223905	0.1646 (−0.5355, 0.8647)
	0.286040	0.4105 (−0.2508, 1.0717) 0.223905	0.644961
Q3 (34.9–38.2 cm)	0.5626 (−0.0553, 1.1805)	0.7253 (0.0888, 1.3618)	0.0650 (−0.6269, 0.7569)
	0.1074512	0.025664	0.853897
Q4 (38.3–56.3 cm)	2.0124 (1.3862, 3.6387)	2.2681 (1.6093, 2.9269)	1.1328 (0.3968, 1.8688)
	<0.000001	<0.000001	0.002597
*p* for trend	<0.001	<0.001	0.003
Stratified by sex^a^			
Men	0.3246 (0.2494, 0.3998)	0.3625 (0.2839, 0.4411)	0.2749 (0.1824, 0.3674)
	<0.000001	<0.000001	<0.000001
Women	0.1405 (0.0918, 0.1892)	0.1716 (0.1205, 0.2227)	0.1378 (0.0850, 0.1907)
	<0.000001	<0.000001	<0.000001
Stratified by race^a^			
Mexican American	0.1317 (0.0134, 0.2500)	0.1520 (0.0337, 0.2702)	0.1125 (−0.0129, 0.2379)
	0.030053	0.012404	0.079954
Other Hispanic	0.1866 (−0.1161, 0.4893)	0.2341 (−0.0814, 0.5495)	0.1636 (−0.1974, 0.5245)
	0.1228925	0.147985	0.375921
Non‐Hispanic White	0.2200 (0.1490, 0.2911)	0.2413 (0.1671, 0.3155)	0.1802 (0.0979, 0.2625)
	<0.000001	<0.000001	<0.000021
Non‐Hispanic Black	0.2434 (0.1559, 0.3309)	0.2562 (0.1662, 0.3463)	0.2179 (0.1180, 0.3179)
	<0.000001	<0.000001	<0.000026
Non‐Hispanic Asian	0.1275 (0.0471, 0.2079)	0.1976 (0.1086, 0.2848)	0.1425 (0.0440, 0.2410)
	0.002086	0.000018	0.004942
Other race	0.6681 (0.4615, 0.9147)	0.8091 (0.5606, 1.0576)	0.5876 (0.2359, 0.9393)
	<0.000001	<0.000001	0.001678

*Note*: Model 1: No covariates were adjusted. Model 2: Sex, age, and race were adjusted. Model 3: Sex, age, race, waist to hip ratio, glucose, high‐density lipoprotein cholesterol, alanine aminotransferase, triglyceride, hypertension, obesity, median controlled attenuation parameter, and uric acid were adjusted.

Abbreviations: CI, confidence interval; LSM, liver stiffness measurement; MAFLD, metabolic‐associated fatty liver disease; MUAC, mid‐upper arm circumference; Q, quartile.

^a^ In the subgroup analysis stratified by sex or race, models 1–3 were not adjusted for their own stratification variable.

## DISCUSSION

This study is the first population‐based study to explore the association between MUAC and liver steatosis and fibrosis detected by VCTE in adults with MAFLD from the NHANES database. Our results suggest that increased MUAC is associated with increased liver steatosis and fibrosis, especially with liver steatosis. Although some nonlinear relations were observed, these trends still existed in multivariable linear regressions. This finding indicates that MUAC could be used as an easily available and simple evaluation instrument for liver steatosis and fibrosis.

The diagnosis of NAFLD was often accompanied by overweight, obesity, and glucose and lipid metabolism disorders, and the pathogenesis of NAFLD and other metabolic disorders was closely interconnected. Many experts in the field of NAFLD are now calling for a change in its terminology.^[^ ^5^, ^17^ ^]^ Therefore, MAFLD, as a new definition, has been suggested to physicians. In our study, compared with patients with nonobese‐MAFLD, patients with obese‐MAFLD had higher anthropometric parameters, liver CAP, and stiffness values; higher levels of biochemical indicators related to metabolism and inflammation; and younger ages. These results are similar to findings in a study from the Philippines that found that patients with obese‐NAFLD were more likely to be younger; had T2DM, hypertension, dyslipidemia, and metabolic syndrome (MS); and had abnormal metabolic parameters (elevated ALT and AST levels, low‐density lipoprotein cholesterol, HDL‐C, TG, and UA) than patients with lean‐NAFLD.^[^ ^18^ ^]^ It was easy for us to understand that nonobese‐NAFLD was considered to have a more favorable biochemical profile and less severe liver histologic injury.^[^ ^19^, ^20^, ^21^ ^]^ However, another study found no differences in the percentages of diabetes mellitus, MS, abnormal obesity, hypertension, and dyslipidemia between nonobese‐ and obese‐NAFLD.^[^ ^22^ ^]^ In addition, patients without obesity were usually older, which was associated with liver cirrhosis and led to further decreases in weight.^[^ ^23^ ^]^ In our study, we also found that patients with nonobese‐MAFLD were older and had a higher BMI; however, lower liver stiffness values, which might be due to patients without obesity but with fatty liver, have fewer metabolic factors associated with the progression of fibrosis, such as IR or the balance and interaction among bile acids, the intestinal microbiome, and systemic metabolism.^[^ ^21^, ^24^ ^]^

In our study, we observed that MUAC was positively associated with liver steatosis in both sex groups and among different race groups. Generally, BMI or waist to hip ratio is commonly used in evaluating obesity and NAFLD. However, there are limitations in the evaluation of these two parameters, such as cirrhotic ascites and lean‐NAFLD, BMI includes body weight and height (which is somewhat complicated to calculate), and BMI value is weakly associated with visceral and liver fat.^[^ ^25^, ^26^ ^]^ MUAC is a representative index for subcutaneous fat in the upper body and is a reliable screening measure for identifying abnormal regional fat distribution, which is less affected by fluid retention.^[^ ^27^, ^28^ ^]^. Although no previous studies have directly proposed a relationship between MUAC and liver steatosis in patients with MAFLD, some researchers have illustrated that a larger MUAC is significantly associated with central obesity and MS.^[^ ^9^, ^29^, ^30^, ^31^, ^32^ ^]^ In addition, MUAC has been commonly used as a simple tool for estimating nutritional status and sarcopenia; patients with sarcopenia had a significantly lower MUAC compared with those without sarcopenia.^[^ ^11^, ^32^, ^33^ ^]^ Therefore, MUAC is closely associated with the metabolic profile of the human body and might be a relatively simple indicator for assessment of liver steatosis in patients with MAFLD.

Clinical factors known to be independently predicative of fibrosis in nonalcoholic steatohepatitis include age, obesity, and T2DM.^[^ ^34^ ^]^ In line with this, we found that liver stiffness in patients with MAFLD increased with an increase in BMI. This is a new topic for exploring the association between MUAC and liver fibrosis in patients with MAFLD. In our study, we further found that MUAC (especially MUAC ≥ 42.0 cm) was positively associated with liver stiffness in patients with MAFLD in both men and women and among non‐Hispanic individuals. At present, there is no published evidence to document a direct relation between MUAC and liver fibrosis in patients with MAFLD. An earlier study investigated whether peripheral and/or abdominal adipose depot size correlated with stage of liver fibrosis in patients with NAFLD and found that men with smaller extremity sizes (*z* scores of MUAC and hip circumference) and premenopausal women with larger extremity sizes were more likely to have more severe fibrosis.^[^ ^35^ ^]^ However, another study found that the arm fat index, a parameter that reflects upper body fat, was negatively associated with liver fibrosis in patients with NAFLD.^[^ ^36^ ^]^ Therefore, controversies in the relationship between MUAC and liver fibrosis in MAFLD still exist, and the representative significance of MUAC for liver fibrosis needs to be assessed in a larger MAFLD population.

In conclusion, MUAC was independently associated with liver steatosis and fibrosis (especially MUAC ≥ 42.0 cm) in patients with MAFLD and might be a simple and convenient tool in the evaluation of MAFLD. In the future, we need to measure MUAC routinely in the outpatient clinic; this will be helpful in the preliminary assessment of the degree of liver steatosis and fibrosis and in the rapid diagnosis and treatment of patients with MAFLD.

## CONFLICTS OF INTEREST

Lai Wei has received research grants from Abbvie, Bristol‐Myers Squibb, and Gilead, and served as a consultant for Gilead, Huahui, MSD, Pfizer, a speaker for Ascletis Pharma, Bristol‐Myers Squibb, Gilead and Kaiyin. The other authors have nothing to report.

## Supporting information


**Appendix S1** Supplementary Information
