# Supplementary material for: Mid‐upper arm circumference is associated with liver steatosis and fibrosis in patients with metabolic‐associated fatty liver disease: A population based observational study
Source: Hepatol Commun. 2022 May 13;6(9):2262–72. doi: 10.1002/hep4.1990 (PMC9426403; doi:10.1002/hep4.1990)
Supplement: Supplementary file 1 — Appendix S1 Supplementary Information [file HEP4-6-2262-s001.docx]

**Table S1 Baseline Characteristics of MAFLD patients with other liver disease assessed by VCTE in the NHANES database, 2017-2018.**

| **Variables** | **nonobese-MAFLD with other liver disease**  **(N=33)** | **obese-MAFLD with other liver disease (N=724)** | | | | **P value** |
| --- | --- | --- | --- | --- | --- | --- |
|  | **BMI**  **(<25 kg/m^2^)** | **BMI**  **(25-29.9 kg/m^2^) N=241** | **BMI**  **(30-34.9 kg/m^2^) N=238** | **BMI**  **(35-39.9 kg/m^2^) N=130** | **BMI**  **(≥40 kg/m^2^) N=115** |  |
| **Age, (year)** | 52.1 ± 13.2 | 48.5 ± 15.3 | 46.0 ± 15.5 | 43.9 ± 14.8 | 41.1 ± 13.6 | <0.001 |
| **Gender, n (%)** |  |  |  |  |  | <0.001 |
| Male | 12 (36.4) | 130 (53.9) | 135 (56.7) | 64 (49.2) | 34 (29.6) |  |
| Female | 21 (63.6) | 111 (46.1) | 103 (43.3) | 66 (50.8) | 81 (70.4) |  |
| **Race, n (%)** |  |  |  |  |  | <0.001 |
| Mexican American | 6 (18.2) | 55 (22.8) | 55 (23.1) | 37 (28.5) | 23 (20) |  |
| Other Hispanic | 1 (3.0) | 36 (14.9) | 35 (14.7) | 9 (6.9) | 6 (5.2) |  |
| Non-Hispanic White | 10 (30.3) | 80 (33.2) | 80 (33.6) | 41 (31.5) | 39 (33.9) |  |
| Non-Hispanic Black | 6 (18.2) | 36 (14.9) | 39 (16.4) | 33 (25.4) | 39 (33.9) |  |
| Non-Hispanic Asian | 7 (21.2) | 24 (10.0) | 14 (5.9) | 2 (1.5) | 0 (0) |  |
| Other races | 3 (9.1) | 10 (4.1) | 15 (6.3) | 8 (6.2) | 8 (7) |  |
| **Anthropometric parameters** |  |  |  |  |  |  |
| BMI (kg/m^2^) | 23.0 ± 1.8 | 27.8 ± 1.4 | 32.4 ± 1.3 | 37.0 ± 1.3 | 45.8 ± 6.1 | <0.001 |
| MUAC (cm) | 28.6 ± 2.5 | 32.5 ± 2.3 | 36.0 ± 2.6 | 38.8 ± 2.9 | 42.7 ± 4.0 | <0.001 |
| Waist circumference (cm) | 87.5 ± 7.4 | 97.6 ± 6.6 | 107.9 ± 7.0 | 116.8 ± 7.4 | 130.8 ± 11.5 | <0.001 |
| Hip circumference (cm) | 95.7 ± 6.3 | 102.7 ± 5.3 | 111.0 ± 5.3 | 120.0 ± 7.0 | 137.4 ± 12.6 | <0.001 |
| Waist-to-hip ratio | 0.9 ± 0.1 | 1.0 ± 0.1 | 1.0 ± 0.1 | 1.0 ± 0.1 | 1.0 ± 0.1 | <0.001 |
| **VCTE parameters** |  |  |  |  |  |  |
| CAP, (dB/m) | 285.0 ± 31.6 | 292.6 ± 29.9 | 312.0 ± 39.5 | 317.2 ± 39.6 | 330.7 ± 45.9 | <0.001 |
| Stiffness, (kPa) | 5.5 ± 2.2 | 5.5 ± 2.7 | 5.9 ± 2.9 | 6.1 ± 3.1 | 10.1 ± 11.9 | <0.001 |
| **Serum test** |  |  |  |  |  |  |
| FPG (mmol/L) | 5.6 ± 1.1 | 5.4 ± 1.1 | 5.8 ± 2.0 | 5.9 ± 2.1 | 5.9 ± 1.8 | 0.051 |
| TC (mmol/L) | 5.3 ± 1.0 | 5.1 ± 1.1 | 5.1 ± 1.1 | 5.0 ± 1.1 | 4.8 ± 1.0 | 0.151 |
| TG (mmol/L) | 1.8 ± 1.3 | 2.0 ± 1.9 | 2.1 ± 1.5 | 2.0 ± 1.3 | 1.7 ± 1.0 | 0.313 |
| HDL-C (mmol/L) | 1.6 ± 0.7 | 1.3 ± 0.4 | 1.3 ± 0.4 | 1.2 ± 0.3 | 1.2 ± 0.3 | <0.001 |
| TBIL (umol/L) | 8.6 ± 4.5 | 7.6 ± 4.0 | 7.5 ± 4.0 | 6.8 ± 4.1 | 6.4 ± 3.6 | 0.013 |
| ALB (g/L) | 41.5 ± 2.7 | 41.0 ± 3.3 | 40.6 ± 3.3 | 39.6 ± 3.2 | 38.5 ± 3.6 | <0.001 |
| ALT (U/L) | 27.1 ± 18.9 | 26.8 ± 19.1 | 30.0 ± 23.5 | 31.5 ± 24.7 | 27.3 ± 23.1 | 0.264 |
| AST (U/L) | 32.5 ± 27.3 | 24.3 ± 13.6 | 24.6 ± 14.6 | 24.4 ± 14.7 | 21.9 ± 14.7 | 0.014 |
| GGT (U/L) | 56.5 ± 57.7 | 43.9 ± 63.7 | 46.8 ± 58.2 | 37.2 ± 31.5 | 37.8 ± 40.4 | 0.225 |
| ALP (U/L) | 82.2 ± 21.1 | 79.2 ± 27.9 | 80.7 ± 24.0 | 84.7 ± 23.2 | 81.8 ± 19.0 | 0.341 |
| Creatinine (umol/L) | 71.9 ± 22.7 | 75.2 ± 31.8 | 77.3 ± 21.1 | 74.4 ± 19.0 | 69.3 ± 18.6 | <0.001 |
| UA (umol/L) | 319.8 ± 71.8 | 325.0 ± 89.1 | 346.3 ± 90.3 | 356.5 ± 92.3 | 343.2 ± 91.2 | 0.007 |
| HbA1c (%) | 5.7 ± 0.7 | 5.7 ± 0.8 | 5.8 ± 1.0 | 6.0 ± 1.2 | 6.0 ± 1.2 | 0.016 |
| **Metabolic diseases** |  |  |  |  |  |  |
| hypertension, n (%) | 10 (30.3) | 56 (23.2) | 45 (18.9) | 29 (22.3) | 29 (25.2) | 0.672 |
| Diabetes, n (%) | 3 (9.1) | 29 (12.0) | 34 (14.3) | 20 (15.4) | 19 (16.5) | 0.428 |
| Pre-Diabetes, n (%) | 3 (9.1) | 24 (10.0) | 38 (16.0) | 21 (16.2) | 18 (15.7) | 0.261 |
| Gout, n (%) | 1(3.0) | 10 (4.1) | 17 (7.1) | 11 (8.5) | 6 (5.2) | 0.659 |
| CHD, n (%) | 0 (0.0) | 6 (2.5) | 3 (1.3) | 2 (1.5) | 3 (2.6) | 0.862 |
| Stroke, n (%) | 0 (0.0) | 13 (5.4) | 7 (2.9) | 3 (2.3) | 2 (2.6) | 0.561 |
| Thyroid problem, n (%) | 3 (9.1) | 21 (8.7) | 25 (10.5) | 7 (5.4) | 11 (9.6) | 0.781 |
| **Sleep time (hours)** |  |  |  |  |  |  |
| Weekdays | 7.4 ± 1.8 | 7.5 ± 1.5 | 7.5 ± 1.6 | 7.4 ± 1.7 | 7.3 ± 1.5 | 0.714 |
| Weekends | 8.1± 2.1 | 8.2 ± 1.5 | 8.4 ± 1.6 | 8.3 ± 1.8 | 8.5 ± 2.0 | 0.333 |
| Trouble Sleep, n (%) | 9 (27.3) | 55 (22.8) | 73 (30.7) | 49 (37.7) | 44 (38.3) | 0.010 |
| **Weight Loss, n (%)** | 24 (72.7) | 160 (66.4) | 161 (67.6) | 97 (74.6) | 71 (61.7) | 0.259 |
| **Medicine, n (%)** |  |  |  |  |  |  |
| Hypertension | 10 (30.3) | 64 (26.6) | 65 (27.3) | 31 (23.8) | 32 (27.8) | 0.053 |
| Female hormones | 5 (15.2) | 21 (8.7) | 19 (8.0) | 4 (3.1) | 11 (9.6) | <0.001 |
| Low dose aspirin | 10 (30.3) | 79 (32.8) | 76 (31.9) | 42 (32.3) | 40 (34.8) | 0.643 |
| **Ratio of Family income to poverty** |  |  |  |  |  | 0.724 |
| ＜1.0 | 5 (15.2) | 35 (14.5) | 39 (16.4) | 21 (16.2) | 26 (22.6) |  |
| 1.0 to <2.0 | 6 (18.2) | 66 (27.4) | 60 (25.2) | 36 (27.7) | 23 (20.0) |  |
| 2.0 to <3.0 | 5 (15.2) | 32 (13.3) | 31 (13.0) | 24 (18.5) | 18 (15.7) |  |
| 3.0 to <5.0 | 9 (27.3) | 36 (14.9) | 46 (19.3) | 20 (15.4) | 22 (19.1) |  |
| ≥5.0 | 5 (15.2) | 43 (27.8) | 41 (17.2) | 15 (11.5) | 16 (13.9) |  |
| NA | 3 (9.1) | 29 (12.0) | 21 (8.8) | 14 (10.8) | 10 (8.7) |  |

**Note**: Continuous variables are shown as mean ± standard deviation (SD). Categorical values are shown as n (%).

**Abbreviations**: ALB, albumin; ALP, alkaline phosphatase; ALT, alanine aminotransferase; AST, aspartate aminotransferase; BMI, body mass index; CHD, Coronary Heart Disease; FPG, fasting plasma glucose; GGT, γ-glutamyl transpeptidase; HbA1c, glycosylated hemoglobin; HDL-C, high density lipoprotein-cholesterol; MAFLD, Metabolic-associated fatty liver disease; MUAC, mid-upper arm circumference; TC, total cholesterol; TG, triglyceride; UA, uric acid; TBIL, total bilirubin; VCTE, Vibration Controlled Transient Elastography (VCTE).

**Table S2 Multivariate analysis for the relationship between MUAC and liver steatosis in MAFLD patients with other liver disease (CAP dB/m)**

|  | **Model 1, β (95%CI)**  **p value (N=757)** | **Model 2, β (95%CI)**  **p value (N=757)** | **Model 3, β (95%CI)**  **p value (N=757)** |
| --- | --- | --- | --- |
| **Baseline MUAC** | 3.3513 (2.7722, 3.9304)  <0.000001 | 3.8492 (3.2441, 4.4542)  <0.000001 | 2.4987 (1.8371, 3.1604)  <0.000001 |
| **Quartiles of MUAC** |  |  |  |
| Q1 (22.5-32.0cm) | **Reference** | **Reference** | **Reference** |
| Q2 (32.1-34.8cm) | 10.1639 (2.0138, 18.3134)  0.014741 | 12.2073 (4.0928, 20.3199)  0.003285 | 11.0321 (3.2244, 13.8398)  0.005757 |
| Q3 (34.9-38.2cm) | 26.5538 (18.6055, 34.5022)  <0.000001 | 27.5059 (19.3433, 35.6685)  <0.000001 | 17.8131 (9.8817, 25.7445)  0.000012 |
| Q4 (38.3-56.3cm) | 38.9848 (31.0023, 46.9673)  <0.000001 | 45.0073 (36.5927, 53.4219)  <0.000001 | 27.8495 (19.2285, 36.4705)  <0.000001 |
| p for trend | <0.001 | <0.001 | <0.001 |
| **Stratified by gender** |  |  |  |
| Men | 4.4422 (3.4619, 5.4226)  <0.000001 | 5.5532 (4.5366, 6.5697)  <0.000001 | 3.7379 (2.6379, 4.8018)  <0.000001 |
| Women | 2.5115 (1.8061, 3.2168)  <0.000001 | 2.8971 (2.1568, 3.6374)  <0.000001 | 1.6706 (0.8381, 2.5031)  0.000100 |
| **Stratified by race** |  |  |  |
| Mexican American | 3.5397 (2.2418, 4.8175)  <0.000001 | 3.8455 (2.4975, 5.1935)  <0.000001 | 2.9040 (1.4540, 4.3541)  0.000128 |
| Other Hispanic | 4.7545 (2.3234, 7.1855)  0.000242 | 5.7375 (3.2311, 8.2439)  0.000023 | 4.0697 (1.5103, 6.6291)  0.002616 |
| Non-Hispanic White | 3.6567 (2.6603, 4.6531)  <0.000001 | 3.9651 (2.9088, 5.0214)  <0.000001 | 2.2710 (1.0535, 2.4886)  0.000316 |
| Non-Hispanic Black | 2.9188 (1.9128, 3.9248)  <0.000001 | 3.0864 (2.0422, 4.1306)  <0.000001 | 2.5211 (1.3580, 3.6842)  0.000039 |
| Non-Hispanic Asian | 4.7483 (2.0406, 7.4560)  0.001276 | 4.1730 (10.6751, 7.6709)  0.024091 | 1.9929 (-1.8945, 5.8803)  0.322092 |
| Other race | 2.9336 (0.6921, 5.1750)  0.013972 | 3.1369 (0.8308, 5.4430)  0.011018 | 3.4353 (0.7958, 6.0749)  0.015901 |

Model 1: No covariates were adjusted.

Model 2: Gender, age and race were adjusted.

Model 3: Gender, age, race, waist to hip ratio, FPG, HDL-C, ALT, TG, UA, hypertension, obesity, and median liver stiffness were adjusted.

In the subgroup analysis stratified by gender or race, model 1, 2 and 3 were not adjusted for stratification variable itself.

**Table S3 Multivariate analysis for the relationship between MUAC and liver fibrosis in MAFLD patients with other liver disease (LSM kPa)**

|  | **Model 1, β (95%CI)**  **p value (N=757)** | **Model 2, β (95%CI)**  **p value (N=757)** | **Model 3, β (95%CI)**  **p value (N=757)** |
| --- | --- | --- | --- |
| **Baseline MUAC** | 0.4789 (0.3749, 0.5783)  <0.000001 | 0.5158 (0.4112, 0.6259)  <0.000001 | 0.4523 (0.3259, 0.5758)  <0.000001 |
| **Quartiles of MUAC** |  |  |  |
| Q1 (22.5-32.0cm) | **Reference** | **Reference** | **Reference** |
| Q2 (32.1-34.8cm) | 0.0439 (-1.3500, 1.4378)  0.950792 | 0.1839 (-1.2432, 1.6111) 0.800638 | 0.0727 (-1.4192, 1.5645)  0.923981 |
| Q3 (34.9-38.2cm) | 0.5691 (-0.7904, 1.9285)  0.412213 | 0.6896 (-0.7464, 2.1256)  0.346882 | -0.0375 (-1.5469, 1.4899)  0.961655 |
| Q4 (38.3-56.3cm) | 5.0721 (3.7068, 6.6374) <0.000001 | 5.3440 (3.8637, 6.8243) <0.000001 | 3.8511 (2.1914, 5.5107) 0.000006 |
| p for trend | <0.001 | <0.001 | <0.001 |
| **Stratified by gender** |  |  |  |
| Men | 0.3991 (0.2665, 0.5317)  <0.000001 | 0.4673 (0.3241, 0.6104)  <0.000001 | 0.3347 (0.1626, 0.5068)  0.000163 |
| Women | 0.5357 (0.3887, 0.6826)  <0.000001 | 0.5445 (0.3869, 0.7021)  <0.000001 | 0.5007 (0.3171, 0.6842)  <0.000001 |
| **Stratified by race** |  |  |  |
| Mexican American | 0.0564 (-0.0368, 0.1496)  0.237314 | 0.0917 (-0.0054, 0.1889)  0.065962 | 0.0270 (-0.0762, 0.1320)  0.609190 |
| Other Hispanic | 0.1712 (-0.0518, 0.3942)  0.136045 | 0.2271 (-0.0087, 0.4629)  0.062551 | 0.0865(-0.1841, 0.3571)  0.2532875 |
| Non-Hispanic White | 0.6316 (0.4356, 0.8276)  <0.000001 | 0.6512 (0.4400, 0.8624)  <0.000001 | 0.6309 (0.3697, 0.8291)  0.000004 |
| Non-Hispanic Black | 0.1674 (0.0797, 0.2551)  0.000261 | 0.1968 (0.1094, 0.2842)  0.000020 | 0.1903 (0.0821, 0.2985)  0.000004 |
| Non-Hispanic Asian | 0.2108 (-0.0211, 0.4426)  0.081499 | 0.2885 (0.0054, 0.5715)  0.052112 | 0.2035(-0.1447, 0.5517)  0.260020 |
| Other race | 0.8270 (0.2970, 1.3571)  0.003866 | 0.8598 (0.2549, 1.4647)  0.008121 | 0.0196 (-0.6959, 0.7352)  0.957448 |

Model 1: No covariates were adjusted.

Model 2: Gender, age and race were adjusted.

Model 3: Gender, age, race, waist to hip ratio, glucose, HDL-C, ALT, TG, hypertension, obesity, median CAP and UA were adjusted.

In the subgroup analysis stratified by gender or race, model 1, 2 and 3 were not adjusted for stratification variable itself.

**
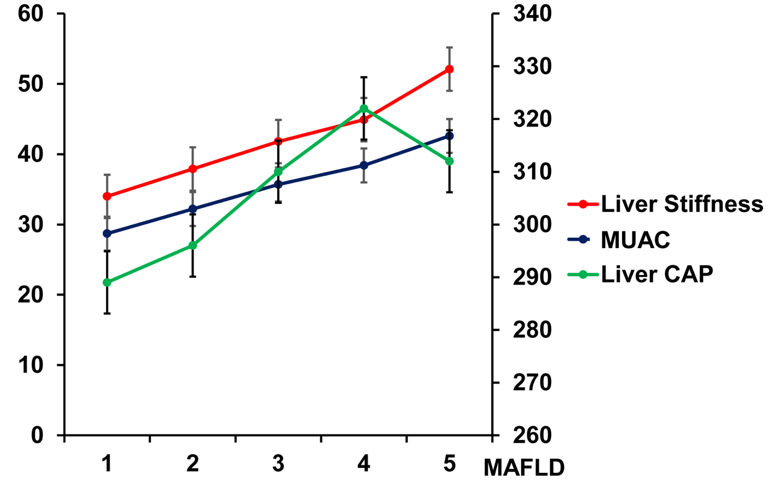
**

**Figure S1. The changing trends of MUAC, liver CAP and stiffness value with the increase of BMI in MAFLD patients.** Red, green and blue lines represented liver stiffness, CAP value and MUAC respectively.


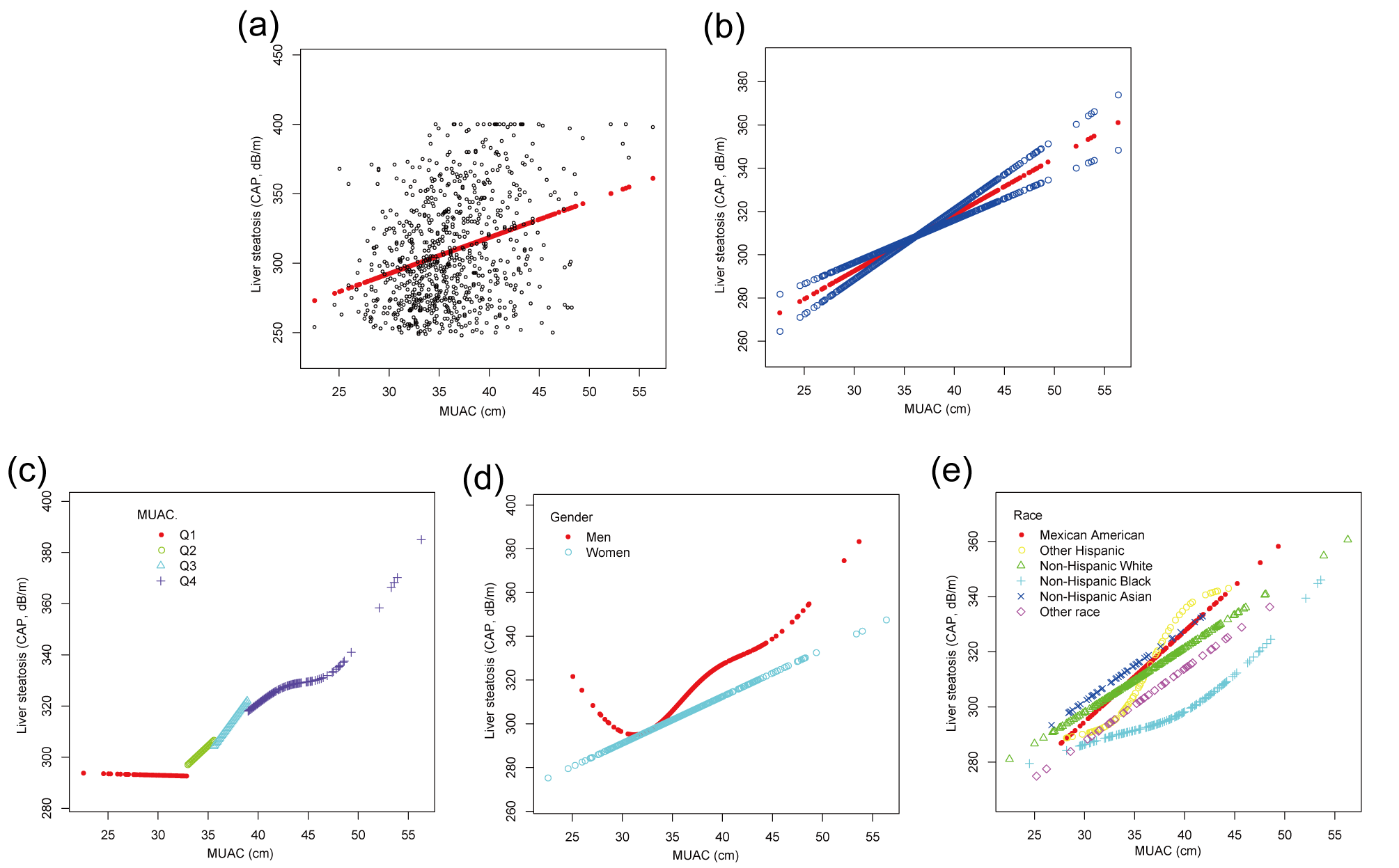


**Figure S2. The association between mid-upper arm circumference (MUAC) and liver steatosis (CAP) in MAFLD patients with other liver disease**. (a) Each sample was represented by a black point. (b) The smooth curve fit (red band) and the 95% of confidence interval (CI) from the fit (blue band) between two variables. Age, gender and race, hypertension, waist to hip ratio, the level of FPG, ALT, HDL-C, TG, and UA, obesity and median liver stiffness were adjusted. The association between MUAC and liver steatosis in (c) four quartiles of MUAC, (d) men and women and (e) different racial subgroups.


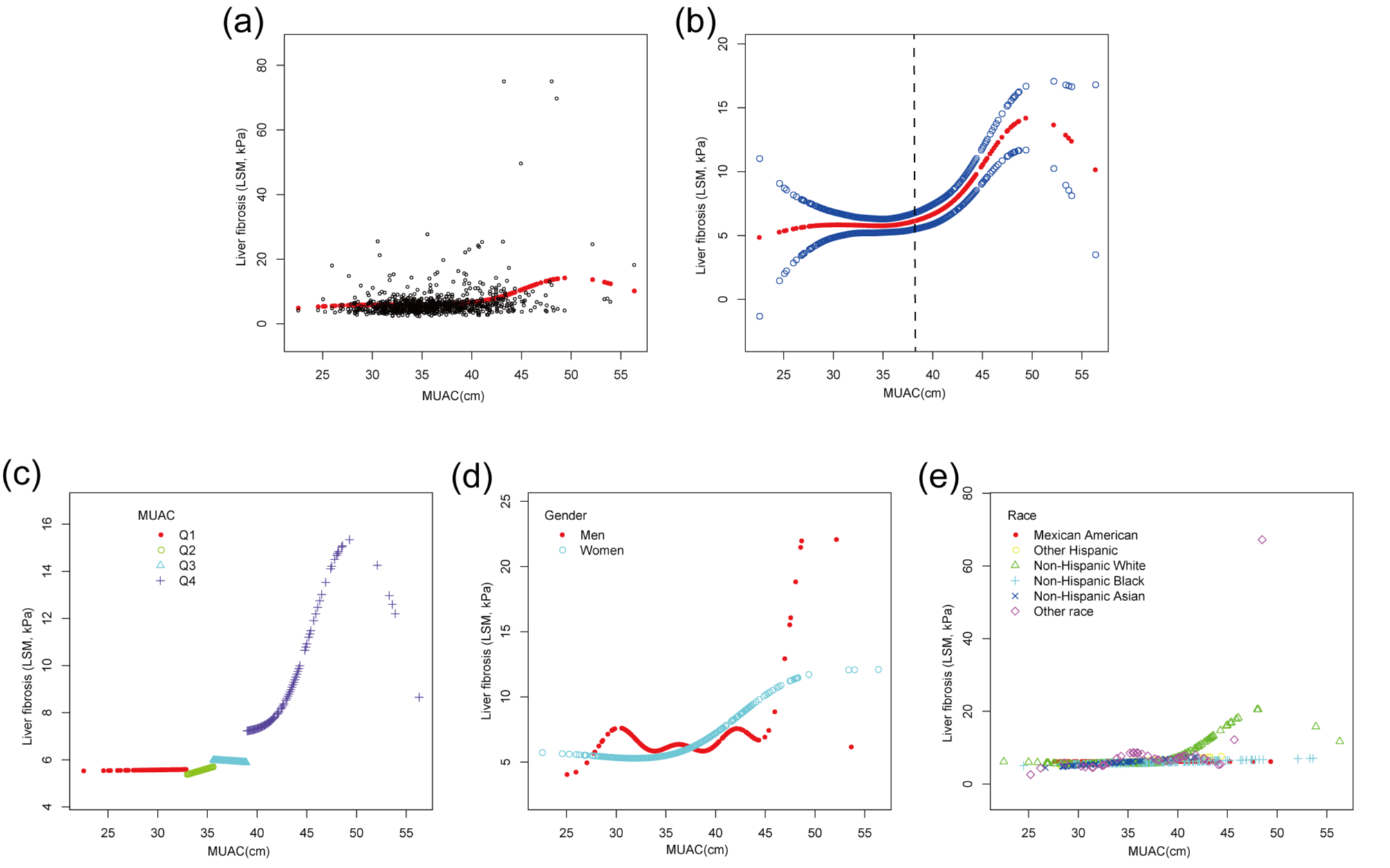


**Figure S3. The association between mid-upper arm circumference (MUAC) and liver fibrosis (LSM, kPa) in MAFLD patients with other liver disease**. (a) Each sample was represented by a black point. (b) The smooth curve fit (red band) and the 95% of confidence interval (CI) from the fit (blue band) between two variables. Age, gender and race, hypertension, waist to hip ratio, the level of FPG, ALT, HDL-C, TG, and UA, obesity, median liver CAP were adjusted. The association between MUAC and liver fibrosis in (c) four quartiles of MUAC, (d) men and women and (e) six racial subgroups.
